# From womb to words: the sex-specific interplay of fetal sex hormones and maternal mood on infant language development

**DOI:** 10.3389/fendo.2026.1817292

**Published:** 2026-06-16

**Authors:** Michaela Reimann-Ayiköz, Jasmin Preiß, Eva Reisenberger, Cristina Florea, Monika Angerer, Manuel Schabus, Dietmar Roehm, Gesa Schaadt, Claudia Männel

**Affiliations:** 1 Centre for Cognitive Neuroscience Salzburg (CCNS), University of Salzburg, Salzburg, Austria; 2 Research Group Neurobiology of Language, Department of Linguistics, University of Salzburg, Salzburg, Austria; 3 Laboratory for Sleep, Cognition and Consciousness Research, Department of Psychology, University of Salzburg, Salzburg, Austria; 4 Department of Geriatric Medicine, Salzburg Universital Hospital Christian-Doppler-Klinik, Salzburg, Austria; 5 Department of Neuropsychology, Max Planck Institute for Human Cognitive and Brain Sciences, Leipzig, Germany; 6 Department of Education and Psychology, Freie Universität Berlin, Berlin, Germany; 7 Department of Audiology and Phoniatrics, Charité – Universitätsmedizin Berlin, Berlin, Germany

**Keywords:** dehydroepiandrosterone (DHEA), hormone, language acquisition, maternal mood, postpartum

## Abstract

**Introduction:**

Language development is influenced by biological and environmental factors, including infant hormonal status and maternal mental health. Previous research on the role of infant sex hormones in language development focused on estradiol and testosterone, yet first evidence indicates that dehydroepiandrosterone (DHEA), the dominant fetal steroid hormone, may be a more sensitive biomarker for language development by shaping the organization of the developing brain. Concerning infants’ language-learning environment, maternal well-being is a key factor, with maternal depressed mood postpartum, even at subclinical levels, negatively affecting language development, as depressed mothers engage less with their children and use less infant-directed speech. The present study examined the interplay of fetal DHEA levels and maternal mood at eight weeks postpartum on receptive language abilities at 12 months in boys and girls.

**Methods:**

Fetal DHEA levels were extracted from hair samples collected two weeks after birth (*n* = 58; 28 girls), allowing fetal hormone milieu quantification in the third trimester. Maternal mood in the subclinical depression range was assessed using the Edinburgh Postnatal Depression Scale. Children’s receptive language abilities were assessed using the German version of the Bayley Scales of Infant and Toddler Development.

**Results:**

Stepwise multiple linear regression analysis revealed fetal DHEA to predict language development in boys, with the effect depending on maternal mood. Only when mothers experienced better mood postpartum were higher DHEA levels related to lower language ability. By contrast, in girls, only maternal mood significantly contributed to language ability, with better mood relating to higher language outcome.

**Discussion:**

Our findings suggest that the effect of infant sex hormones on language development follows sex-specific patterns and appears to be modulated by the learning environment. Moreover, our results emphasize the importance of mental support during the early stages of language development.

## 1 Introduction

Language acquisition in early childhood is a complex, multifaceted process shaped by both biological and environmental factors, drawing research interest since the 19th century within the nature-nurture debate. The ‘nature’ perspective highlights the role of genes and hormones in building the neurobiological foundation of early language abilities (1–5). In the ‘nurture’ perspective, social interaction, exposure to language, and responsive caregiving play a critical role in shaping and stimulating these capacities (6–11). The roles of nature and nurture are increasingly recognized as a dynamic interplay: epigenetic programming allows environmental signals to modulate gene expression (12, 13), while hormonal activity is also considered to be responsive to environmental conditions (14, 15). Therefore, it is essential to understand how biological and environmental factors interact in shaping early language development (16–18).

Regarding biological foundations of language development, there is growing interest in the role of fetal and infant sex hormones as biomarkers for language ability, as they have been reported to affect language processing by shaping the development of the brain’s functional lateralization, hemispheric organization, and brain maturation (19–22). Research into sex hormones has mainly focused on fetal and infant estradiol and testosterone, with opposite effects on language development: High estradiol levels have been demonstrated to be positively related to language ability (4, 5, 23), whereas high testosterone levels have been primarily found to be negatively associated with language ability (4, 24–26), although for girls this association has also been reported to be positive (27) or non-linear (28). Thus, initial evidence suggests that testosterone and estradiol influence early language development, yet studies vary in methodology and timing of hormone extraction and demonstrate sex-related differences in predictive power and direction of effects (24, 25, 27–29).

Turning attention to testosterone’s and estradiol’s shared precursor hormone dehydroepiandrosterone (DHEA), though largely unexplored (30, 31), may represent a promising candidate for understanding early neurodevelopment, as it is considered to play a crucial role in the organization of the fetal brain (32–35). In the fetus, DHEA is the most prevalent steroid hormone, alongside its sulfate ester, DHEA-S (referred to jointly as ‘DHEA(S)’, unless otherwise specified) (36, 37). To explain how DHEA(S) may be linked to neurodevelopment outcomes, it is essential to explore the mechanistic pathways through which the neurosteroid may act in the fetal organism, with evidence mostly derived from animal research: Through its proposed anti-glucocorticoid role in the hippocampus, DHEA may inhibit cortisol signaling via enzymatic mechanisms (38–40), potentially limiting cortisol-mediated brain maturation in the third trimester (41, 42). Furthermore, DHEA(S) modulates the gamma-aminobutyric acid type A (GABA_A_) receptor (43, 44), thereby potentially decreasing GABA_A_-mediated neuronal excitability in the developing brain (45, 46). Both pathways may operate differently in males and females (47–50), pointing to a sex-specific vulnerability during fetal brain development.

Building on its mechanistic role during the prenatal stage, elevated levels of DHEA(S) highlight the fetal phase as a critical period for understanding development (51, 52), with concentrations peaking in the third trimester (53). Using newborn hair, hormone levels in the third trimester can be retrospectively assessed, providing a stable, long-term index of fetal endocrine activity, unaffected by circadian or situational variation (54–56). Compared to its sulfate DHEA-S, DHEA is considered to be a more sensitive biomarker of endocrine activity (56, 57). Moreover, by crossing the blood-brain barrier, DHEA exhibits greater bioavailability in the brain, potentially contributing to central nervous system function (37). Accordingly, hair-derived DHEA may serve as a more reliable biomarker of third-trimester endocrine function than DHEA-S.

To our knowledge, only two studies so far have examined DHEA(S) in relation to infant language development: In the study of Lee et al. (58), DHEA-S levels were assessed at birth, mediating the relationship between placental inflammation and cognitive and language development at 12 months of age in children from low socioeconomic backgrounds, with no direct association between DHEA-S and development observed. In our previous study, we provided first evidence that fetal DHEA may serve as a biomarker of language development in boys, with higher DHEA levels indicating poorer language abilities at six months of age (59). This initial evidence points to a critical role of fetal DHEA in language development. However, whether the predictive value of fetal DHEA extends into later developmental stages remains to be established, emphasizing the importance of further studies to clarify its role in language development.

In addition to biological factors, infants’ language-learning environment plays a vital role (60–62), with quantity and quality of speech input (63–66) and caregiver-interactions predicting language outcome (67, 68). In this context, maternal postpartum health plays a fundamental role in shaping the early language learning environment: Even at subclinical levels, postpartum maternal depressive symptoms can negatively affect language development (69, 70). Mothers suffering from postpartum depression are less responsive to their infants (71) and engage less in activities that enrich their infants’ development (72–74), such as reading, playing, and storytelling (75). Depressed mothers not only talk less with their infants and show slower verbal responses (76), but their infant-directed speech is less pronounced as indicated by reduced pitch modulation (76–78), leading to weaker infant responses to maternal speech (79, 80). Given these changes in mother-infant interactions, postpartum maternal depressive symptoms can negatively affect infants’ language development (81–83).

Taken together, both infant sex hormones and maternal postpartum depression were shown to influence early language development. Understanding how these biological and environmental factors interact is essential to gaining a deeper insight into factors shaping early language developmental pathways. Thus, the present study examined how fetal DHEA levels extracted from newborn hair samples and maternal mood at eight weeks postpartum, as well as their interplay, relate to receptive language abilities at 12 months in boys and girls. Specifically, we hypothesized that higher levels of the neurosteroid DHEA would have a negative effect on language development at 12 months in boys only, as sex-specific effects on language development were previously reported for testosterone (25) and DHEA (59). Importantly, higher fetal DHEA levels should not be interpreted as detrimental to development, but rather as reflecting a temporary prioritization of other neurodevelopmental processes during early brain maturation in boys, with early language skills being one of several domains competing for developmental resources at this stage. Moreover, we expected lower maternal mood to be negatively associated with language development in boys and girls, given that maternal depressive symptoms are considered a risk factor for children’s language development (reviewed in 69). Finally, we proposed that fetal DHEA would significantly interact with maternal mood in predicting language development, building on initial evidence that environmental factors interact with DHEA(S)’s influence (58).

## 2 Materials and methods

### 2.1 Participants

The current data were collected as part of a longitudinal project, following 67 mother-infant pairs from 34 weeks of gestation until 12 months postpartum. While 34 weeks of gestation is relatively early, this timing reflected the design of the broader longitudinal study rather than a study-specific rationale. Study approval was granted by the ethics committee of the University of Salzburg (EK-GZ: 12/2013). Mothers provided informed consent prior to participation. Maternal inclusion criteria for the present study were: (1) age of at least 18 years, (2) no pregnancy complications, except for transient findings in *n =* 3 women (i.e., resolved placenta previa; cervical insufficiency without further clinical relevance; hyperemesis and abnormal nuchal translucency with unremarkable follow-up NIPD result), (3) no prenatal smoking or alcohol consumption and (4) German as native language. Infant inclusion criteria were: (1) no developmental delays in cognitive or language domains at 12 months of age according to pediatric evaluation, (2) gestational age of at least 37 weeks, (3) German as the primary environmental language, (4) no hearing impairments, and (5) no family history of language impairments (first- and second-degree). These criteria were confirmed through a study-specific questionnaire, which also gathered demographic information, including maternal education and the relationship status with the child’s biological father (for an overview of demographic and participant characteristics, see **Table 1**).

**Table 1:** Participant and demographic information on final sample (*n =* 58).

Variable	
Characteristic	Value
Maternal and family characteristics	
Maternal age at eight weeks postpartum, mean (SD) [range]	32.94 y (4.56) [21-42]
Maternal relationship status at the 34th GW (%)	
Married to the child’s father	50.00
In a relationship with the child’s father	46.55
Single	3.45
Professional qualification of mothers (%)	
Without vocational qualification	1.72
Completed vocational training	18.97
Higher vocational education	18.97
University degree (or higher)	60.34
Characteristic	Value
Infant characteristics	
Sex (% female)	48.28
Gestational age at birth in weeks, mean (SD) [range]	39 + 6 (7d) [37-42]
Delivery mode (%)	
Vaginal delivery	62.07
Operative vaginal delivery (forceps or vacuum)	12.07
C-section	25.86
Birth weight, mean (SD) [range]	3418 g (406) [2600-4740]
Birth height, mean (SD) [range]	51.93 cm (2.43) [48-58]
Age at hair collection, mean (SD) [range]	16.24 d (2.96) [12-26]
Age at 12 months, mean (SD) [range]	12.28 M (0.38) [10.83-13.30]
Hair mass, mean (SD) [range]	2.33 mg (1.93) [0.2-7.5]
Hair length, mean (SD) [range]*	1.97 cm (0.71) [1.0-3.5]

Maternal age refers to the age at EPDS assessment at eight weeks. Infant age at 12 months refers to the BSID-III assessment at 12 months (* one missing value).

**Figure 1:**
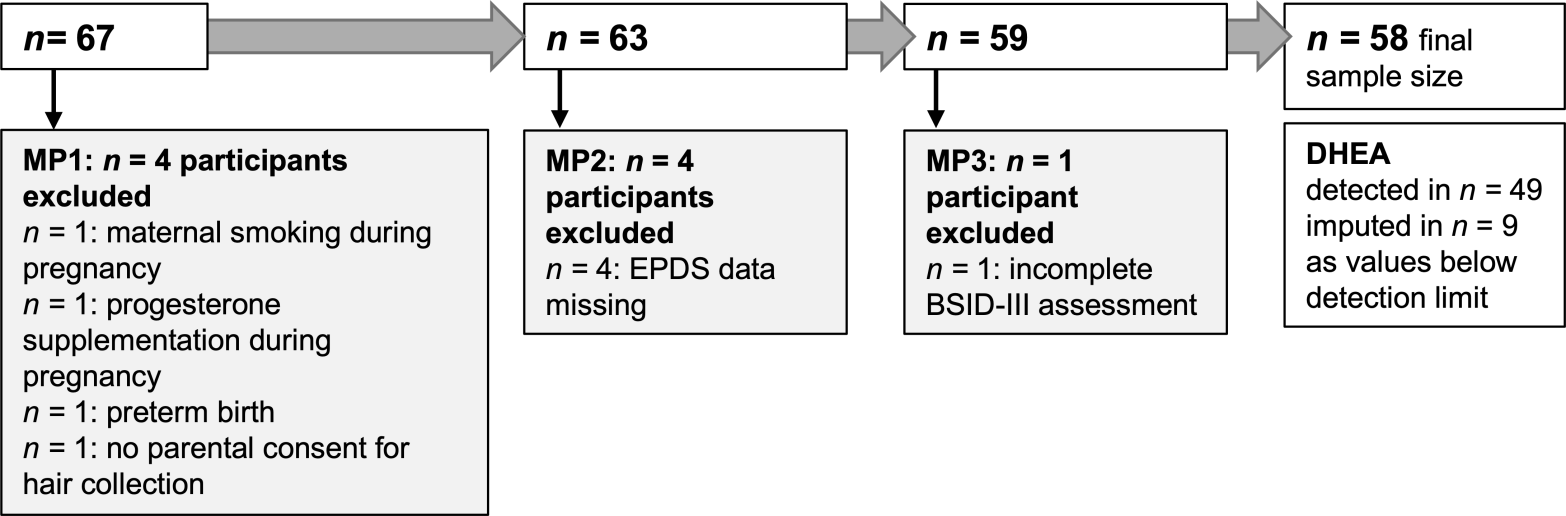
Flow chart of sample inclusion and exclusion. From the initial 67 mother-infant pairs, four infants were excluded from hormone analyses, and data from an additional five was incomplete. The final sample comprised 58 dyads, of which nine fetal DHEA values were below the limit of quantification and thus were imputed for further analysis.

Of the original 67 mother-infant pairs, four pairs were excluded from hair-sample analysis for the following reasons: *n =* 1 with parental decision not to provide any hair samples, *n =* 1 premature birth, *n =* 1 maternal smoking during pregnancy, *n =* 1 maternal progesterone treatment during pregnancy. All other mother-infant pairs with complete datasets were included (*n =* 5 incomplete datasets), with the final participant sample comprising *n =* 58 mother-infant dyads (28 female infants), as illustrated in **Figure 1**. As DHEA levels in *n =* 9 hair samples were below the lower limit of quantification, these values were imputed for further analysis (see method section).

Statistical power of the sample size was evaluated separately for boys (*n =* 30) and girls (*n =* 28), based on previous sex-specific findings on fetal DHEA and language development (59). Sensitivity power analyses were conducted using G*Power with α = .05 and a statistical power of .80. The initially specified regression models included two predictors and their interaction term and thus, the full candidate model comprised three predictors. The analysis showed that large effects could be detected with adequate power (*R^2^* = .30 for boys; *R^2^* = .31 for girls), whereas small to moderate effects may not be reliably detected.

### 2.2 Determination of fetal hair (DHEA)

To quantify fetal DHEA levels, we collected newborn hair two weeks postpartum for hormone analysis (*M_child age_* = 16.24 d, *SD* = 2.96 d). Scalp hair samples were cut at the posterior vertex, wrapped in aluminum foil, and stored dry and protected from light prior to analysis. Full hair strands were analyzed, reflecting the early growth stage, with descriptive statistics for hair length and hair mass provided in **Table 1**. Analysis was performed by the Dresden LabService GmbH at the Technische Universität Dresden, using column-switching liquid chromatography tandem mass spectrometry assay with atmospheric-pressure chemical ionization (LC-APCI-MS/MS) developed by Gao et al. (54): First, hair strands were washed with isopropanol to minimize potential external contamination prior to analysis. DHEA was extracted from the whole, non-pulverized hair via methanol incubation (Carl Roth GmbH & Co. KG; Karlsruhe, Germany). On-line solid phase extraction (SPE) was applied using a column-switching strategy, followed by analyte detection on an AB Sciex API 5000 QTrap mass spectrometer. For stock solutions, DHEA from Sigma-Aldrich (Hamburg, Germany), and for deuterated internal standard solutions DHEA-d4 from Biocrates Life Sciences AG (Innsbruck, Austria) were used. As hair samples were assessed in a single batch, estimates of inter-assay variation were not applicable. The intra-assay coefficient of variation ranged between 9% to 12%, consistent with the Food and Drug Administration’s acceptance threshold of ≤15% (84). The lower limit of detection for DHEA was 1 pg/mg.

### 2.3 Maternal mood

Eight weeks postpartum (*M_maternal age_* = 32.94 y, *SD* = 4.56 y), maternal mood was assessed using the German version (85) of the Edinburgh Postnatal Depression Scale (EPDS, 86), a self-administered screening instrument consisting of ten questions about the mother’s mood over the previous seven days. Each question has four response options on a scale ranging from 0 to 3. The maximum possible score is 30; higher scores indicate higher levels of depressed mood. Since we were interested in subclinical depressive symptoms, our sample consisted exclusively of mothers whose scores were below the clinical cut-off of 13, with scores of 13 or higher indicating a high probability of clinical depression. This cut-off provides high specificity but low sensitivity (87). Accordingly, EPDS scores were used to capture variation in postpartum maternal mood below the clinical cut-off and are therefore referred to as reflecting maternal mood throughout the manuscript.

### 2.4 Infant language development

At 12 months of age (*M_infant age_* = 12.28 months, *SD* = 0.38), the German version of the Bayley Scales of Infant Development (BSID-III, 88–90) was used to assess the children’s receptive language development. The assessment was conducted by a female trained examiner in the presence of one parent. In the German norming study (*N =* 1009), the language scale showed high internal consistency (Cronbach’s α = .86). It consists of two subtests, testing receptive and expressive communication skills, with the raw scores of the receptive subscale employed in the present study, as it seems to be a more reliable indicator of subsequent language development than the expressive subtest (90). The receptive subtest examines the child’s play behavior, responsiveness to speech, use of communicative gestures, and object identification.

### 2.5 Data analysis

Multiple linear regression analysis with stepwise backward selection, implemented via the *MASS* package (91), was conducted to evaluate the main effects of fetal DHEA levels, maternal postpartum mood, as well as their interaction effect, on infant receptive language development at 12 months of age. The analysis was conducted separately in boys and girls, given the previously reported analysis strategies that were based on sex-specific hormonal effects (27). The final selected models are described as the best-fitting models for each sex. To investigate the interaction effect of fetal DHEA and maternal mood, the Johnson-Neyman procedure was applied using the *interactions* package (92) to determine the range of maternal postpartum mood, for which the effects of DHEA on language outcomes at 12 months became statistically significant.

Before running the statistical analyses, multiple imputation was performed for *n =* 9 DHEA values below the quantification limit, following the approach by Herbers et al. (93) using the R package *lnormimp*. This method accounts for the characteristic right-skewness of biological measures, as observed in our sample’s DHEA distribution (*skewness* = 0.80, *z* = 2.50, *p = .*01, Shapiro-Wilk: *W* = .94, *p* <.01), by fitting a log-normal distribution. To avoid potential multicollinearity, the analyses were conducted based on the original scale of DHEA levels. Following recommendations for missing data rates below 30% (94), all analyses were conducted separately for *m* = 20 imputed datasets, with pooled estimates derived using Rubin’s rules (95), as implemented in the *miceadds* package (96).

Given its potential role as an explanatory factor, infant age at the 12-month assessment (*M =* 12.28 months, *SD* = 0.38) was tested for its association with receptive language development outcomes using bivariate Spearman correlations, with no significant correlation found (*p* >.05, see **Supplementary Table 1a**). For fetal DHEA levels, hair length, hair mass, and infant age at hair sample collection were considered as potential methodological cofounders, with no significant correlations observed (*all p* >.05, see **Supplementary Table 1b**). Consequently, these factors were not entered into the regression analyses alongside DHEA levels and maternal mood.

All regression models met the assumption of homoscedasticity, and no multicollinearity was detected (*VIF* < 3.2). Sensitivity analyses were conducted to assess the robustness of the findings, with statistical outliers (*standardized residuals >* 2.5 SD) excluded where applicable (see **Supplementary Table 2b**). Statistical analyses were conducted using RStudio 2025.09.2 + 418 (97), with a significance level of *p = .*05.

## 3 Results

Descriptive statistics on fetal DHEA levels, maternal mood eight weeks postpartum (EPDS), as well as 12-month infant scores on the BSID-III receptive language scale are presented in **Table 2**, with no significant differences between infant sexes (*all p* >.05).

**Table 2:** Descriptive statistics for fetal DHEA levels, maternal mood eight weeks postpartum, and infant language and cognitive development at 12 months.

Variable	Final sample (*n* = 58)	Males (*n* = 30)	Females (*n* = 28)	p-value
Fetal DHEA level in pg/mg^2^	17.68 (13.15) [0.54-55.51]	16.05 (13.39) [0.59-55.51]	19.41 (12.89) [0.54-52.58]	.30
BSID-III Receptive language subscale raw scores^1^	15.19 (2.39) [10-21]	14.97 (2.47) [10-20]	15.43 (2.32) [11-21]	.47
EPDS: Maternal mood at eight weeks postpartum^2^	3.40 (2.91) [0-12]	3.83 (3.34) [0-12]	2.93 (2.34) [0-8]	.47

Mean (SD) and [range] given for each variable, no sex differences in any of the variables observed, to test for sex differences, t-test were used when normally distributed^1^ and Wilcoxon-rank sum test as a non-parametric alternative^2^. For hormone values below the quantification limit (*n =* 9), the mean across *m =* 20 imputed datasets was applied.

**Figure 2:**
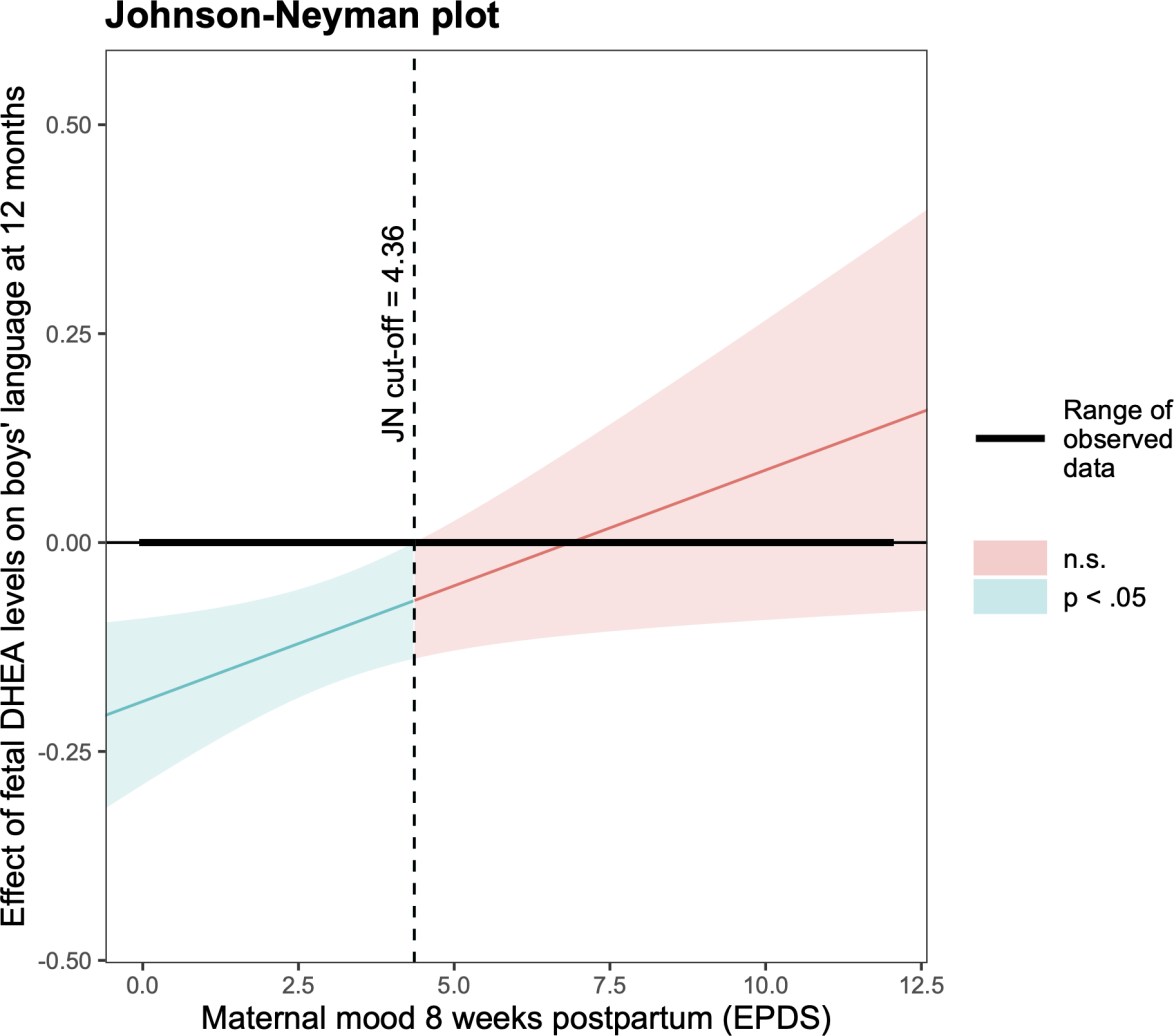
The Johnson-Neyman (JN) plot shows the effect of fetal DHEA on boys’ (*n =* 30) receptive language scores at 12 months, depending on the observed range (0-12) of maternal mood at 8 weeks postpartum. Specifically, below the EPDS cut-off value of 4.36, the green line indicates a significant association (*p* <.05). In contrast, above this cut-off, the red line shows no significant effect.

In boys, stepwise multiple regression analyses identified the model, including fetal DHEA levels, maternal mood, and their interaction effect, as the best-fitting model, significantly predicting receptive language abilities at 12 months of age (*pooled^adj.^ R^2^* = .32, *p <*.01, see **Supplementary Table 2a**). In addition to the main effects of fetal DHEA levels (β = -1.03, *p <*.001) and maternal mood (β = -0.70, *p = .*01), their interaction effect (β = 0.63, *p = .*03) emerged as a significant predictor. To further understand how fetal DHEA levels interact with maternal mood in predicting boys’ language outcome, the Johnson-Neyman analysis was performed, identifying a cut-off point for maternal mood of 4.36 (pooled across *m =* 20 imputations, range = 4.34-4.39, see **Figure 2**).

**Figure 3:**
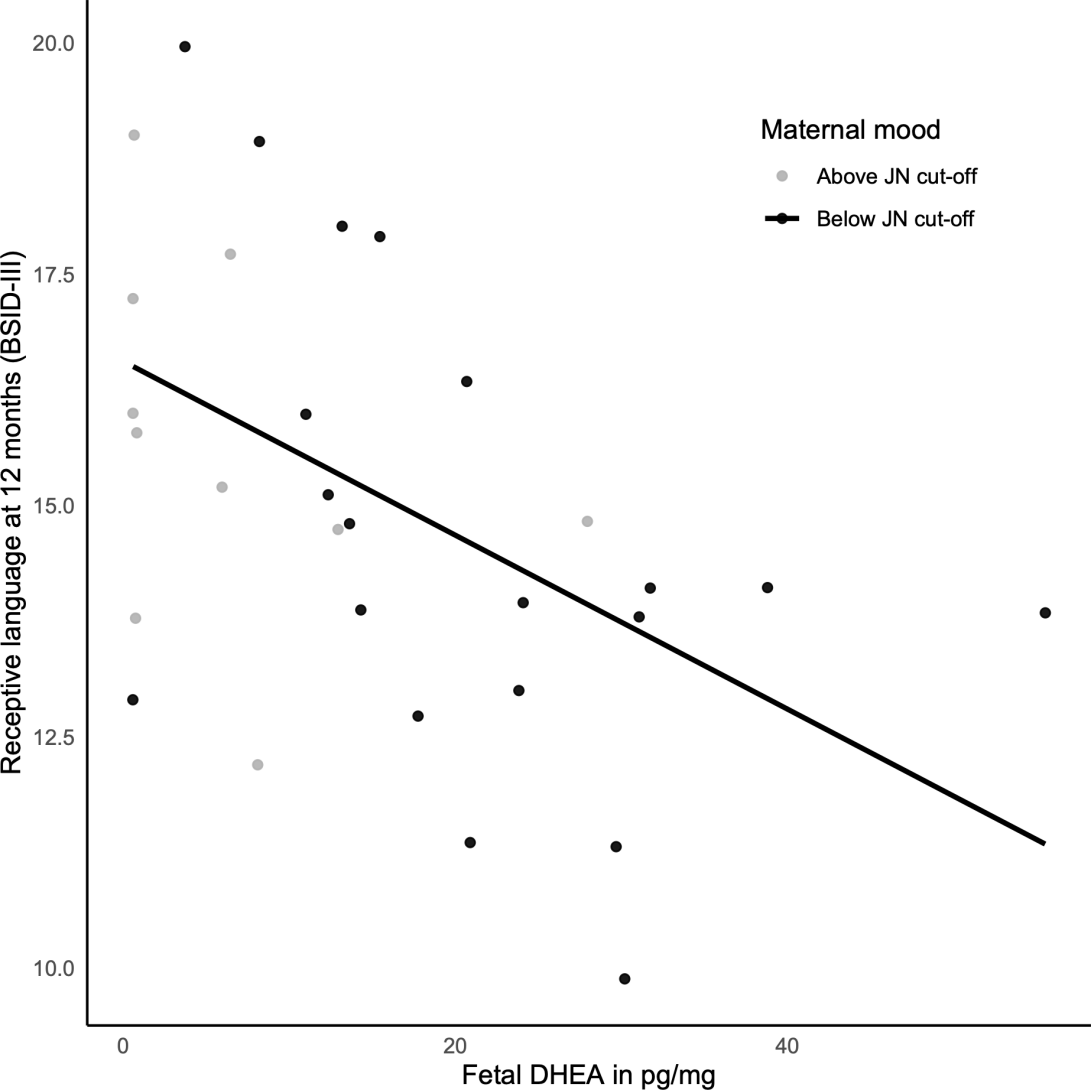
Association between fetal DHEA levels extracted from newborn hair samples and receptive language abilities at 12 months (BSID-III) in boys (*n =* 30, including *n* = 6 imputed DHEA values, mean scores across imputed datasets shown, data jittered), with higher DHEA levels corresponding to lower language abilities (*p <*.001). The effect is moderated by maternal mood (*p* = .03) at eight weeks postpartum, with a significant negative association observed only when maternal mood is higher (JN cut-off: EPDS < 4.36).

This indicates that fetal DHEA significantly predicted boys’ receptive language at 12 months when maternal mood was more positive (EPDS score below 4.36), with higher fetal DHEA levels being associated with lower language outcomes (see **Figure 3**). Sensitivity analyses confirmed these findings (see **Supplementary Table 2b**).

**Figure 4:**
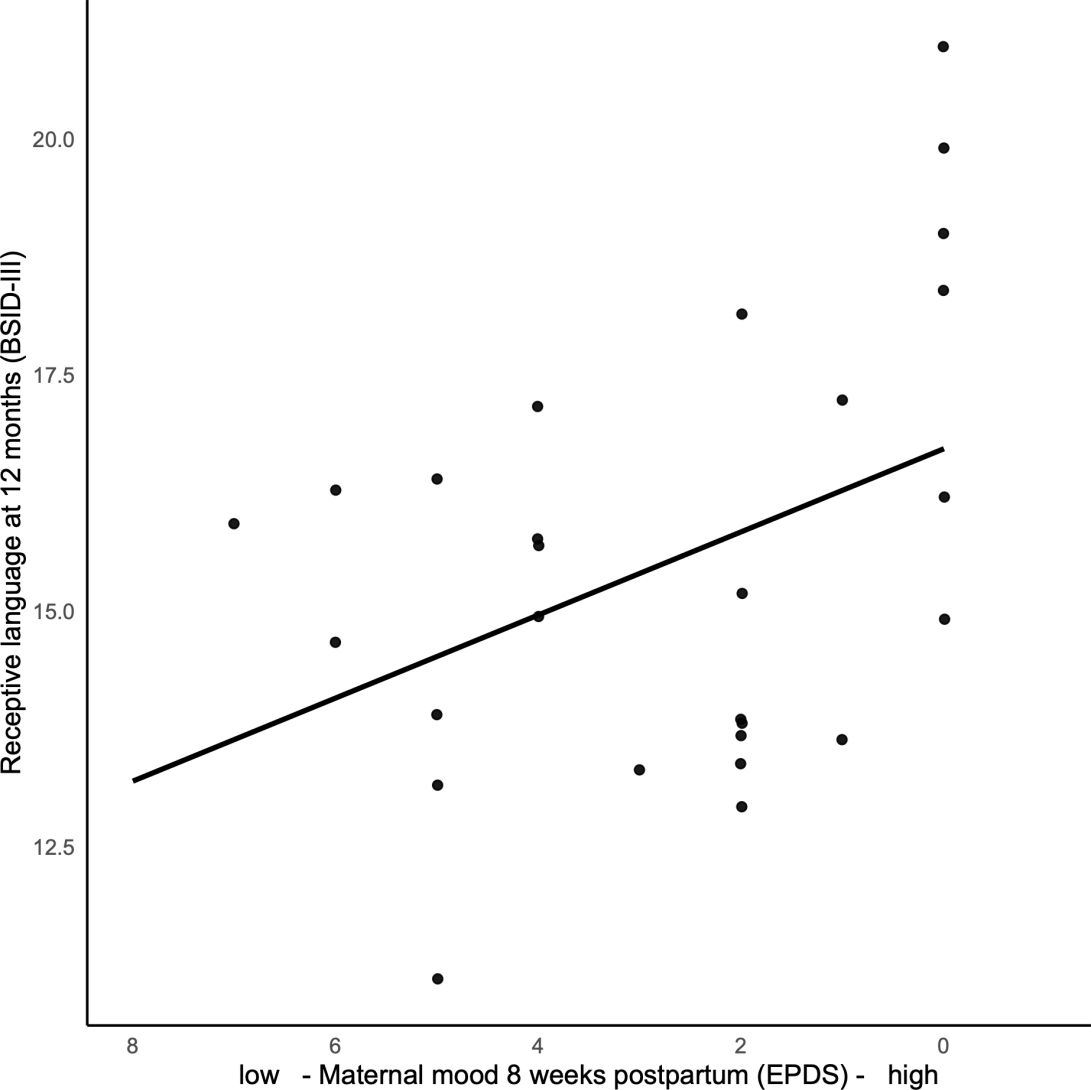
Association between maternal mood at eight weeks postpartum (EPDS) and receptive language abilities at 12 months (BSID-III) in girls (*n =* 28). Note that higher receptive language abilities at 12 months were associated with better maternal mood at eight weeks postpartum (*p* = .02), with lower scores indicating more depressive symptoms (data jittered to prevent overlapping points).

For girls, stepwise multiple regression analysis revealed the model, only including maternal mood at eight weeks postpartum, as the best-fitting model, significantly predicting receptive language abilities at 12 months of age (*pooled^adj.^ R^2^* = .17, *p = .*02, see **Supplementary Table 3**). Here, better maternal postpartum mood was associated with higher receptive language abilities (β = -0.45, *p = .*02, see **Figure 4**).

## 4 Discussion

The present study investigated the predictive value of fetal DHEA levels extracted from newborn hair, maternal mood eight weeks postpartum, as well as their interacting effect on behavioral language outcomes at 12 months of age in boys and girls. Our findings revealed that fetal DHEA negatively predicted receptive language development in boys only, with higher DHEA levels corresponding to lower language ability. However, being moderated by maternal mood, the relationship only reached significance when mothers experienced more positive postpartum mood. In contrast to boys, fetal DHEA showed no predictive value for later language abilities in girls. Instead, maternal mood eight weeks postpartum significantly contributed to girls’ receptive language proficiency at 12 months of age, with better maternal mood being associated with higher language outcome. These findings reveal sex-specific patterns of fetal hormones predicting language development: For boys, fetal DHEA concentrations in the third trimester offer an early biological indicator of infant language abilities. Yet, the effect differs depending on maternal mood, highlighting the complementary impact of the language-learning environment. For girls, fetal DHEA showed no predictive value. Rather, subclinical variations in maternal postpartum mood predicted infant language development.

The present study confirmed fetal DHEA as a promising biological marker of early language development at 12 months of age in boys, but not in girls, extending our previous sex-specific findings of fetal DHEA in boys predicting language development at six months of age (59). To better understand the observed sex-dependent effect of fetal DHEA on language development, it is important to consider its underlying biological mechanisms. Although DHEA is classified within the androgen system, it exerts only weak direct androgenic effects. Rather, fetal DHEA should be understood as both a marker of the prenatal steroidogenic milieu and a neurosteroid capable of modulating early brain development (37), predominantly during sensitive periods such as the third trimester, particularly via GABA_A_-receptor and cortisol-related pathways.

In the fetal brain, activation of GABA_A_-receptors is often depolarizing and can therefore have an excitatory-like effect, unlike its predominantly inhibitory role in the adult brain (45, 46). This early GABA_A_-mediated depolarization supports cortical maturation by promoting dendritic growth, excitatory synapse formation via N-methyl-D-aspartate (NMDA)-receptor activation, and the establishment of early cortical circuits (98, 99; for a review, see 46). NMDA-dependent activity further supports the maturation and integration of GABAergic inhibitory interneurons, which later regulate cortical activity and excitation-inhibition balance (100). Importantly, GABAergic development continues into late gestation with a substantial proportion of GABAergic neurons continuing to migrate toward the cortex (99). Moreover, GABA_A_- and NMDA-dependent signaling may contribute to auditory cortical maturation (101, 102), relevant for later language development (see also 103), although direct evidence from human fetal auditory networks remains limited. Given that DHEA(S) can negatively modulate the GABA_A_-receptor function (43, 44), higher fetal DHEA levels may attenuate GABAergic signaling during fetal maturation. This provides a possible mechanism linking elevated fetal DHEA levels to less optimal language development, potentially by affecting synaptic maturation, excitation-inhibition balance, and auditory network maturation. Notably, GABA_A_-mediated excitatory activity is more pronounced and longer-lasting in males (47), which may account for the observed sex-specific sensitivity. Moreover, DHEA acts as a glucocorticoid antagonist in the hippocampus. Since the late-gestation rise in cortisol supports brain maturation and thereby drives neurodevelopment (41, 42), DHEA may attenuate these effects (39, 40), thereby limiting cortisol-mediated neural development. Mechanistically, fetal DHEA modulates the enzyme 11ß-hydroxysteroid dehydrogenase (11ß-HSD) type 2 (38, 43), responsible for regulating fetal cortisol exposure (104, 105), with placental 11ß-HSD-2 being less active in female compared to male offspring (48). Whereas our data suggest that fetal DHEA has a higher predictive value in boys, first evidence on maternal prenatal cortisol points to the opposite pattern, with stronger effects observed in girls (41, 106, 107), yet studies on fetal cortisol are lacking. Together, these findings point to sex-dependent sensitivity to specific hormonal pathways. Future studies are required on cortisol and DHEA, as well as mechanisms during late gestation to disentangle their respective effects on child development (31, 108).

Interestingly, the effect of fetal DHEA on language development in boys emerged only for cases of more positive mood after birth, indicating that the impact of prenatal hormonal exposure is moderated by the early postnatal environment. However, research on environmental moderators in the context of infant hormones as biomarkers of language development remains scarce. In comparison, Lee et al. (58) reported the effect of DHEA-S on language outcomes to be moderated by household socio-economic status (SES). As the effect was more pronounced among low SES families, the authors suggested it reflects ‘unequal prenatal hormone programming’, with vulnerable groups being particularly impacted. According to the biological sensitivity to context (BSC) hypothesis (109), early biological systems are calibrated to environmental conditions in a non-linear manner. High biological sensitivity may emerge both in highly adverse environments, where heightened reactivity supports vigilance and survival, and in highly supportive environments, where increased plasticity facilitates learning and adaptation. In contrast, moderate environments may promote lower biological sensitivity, as neither heightened vigilance nor maximal plasticity is required. Within this framework, prenatal hormonal influences such as DHEA may exert stronger developmental effects in contexts characterized by greater biological sensitivity, while being attenuated under more average conditions. Turning to our study, mothers varied in postpartum mood from more positive to less positive within the mentally healthy range, and, in line with the BSC framework, the effect was only observed at the higher end of maternal positive mood. As our sample did not include clinically depressed mothers, no conclusion can be drawn for highly adverse conditions. These findings highlight the need to examine the effects of prenatal hormones on development in both low- and high-adversity environments, reflecting the complex interplay between nature and nurture.

Concerning the infant learning environment, we found maternal mood eight weeks postpartum predicting receptive language development at 12 months, with higher maternal mood relating to higher language outcome. Specifically, our findings suggest that language development in girls is modulated by the environment, whereas in boys it seems to be affected by an interplay of hormones and environment. Many mothers face emotional challenges after birth, with about 50% experiencing transient ‘baby blues’ (110) and 10% to 20% (111, 112) suffering from postpartum depression, defined as persistent depressive symptoms occurring within 12 months after childbirth (113). Maternal postpartum depression is well documented as contributing negatively to a child’s language development (69, 82). Even depressive symptoms in the subclinical range are considered to be negatively related to infant language abilities (70), with our study providing further evidence from a non-clinical sample of mothers. These findings underscore the importance of social support during the early stages of family life. In particular, integrating postpartum-depression screening measures into routine pediatric appointments, offering accessible counseling services, and setting up support groups for affected families could significantly improve maternal healthcare and support (114–116).

Although our sample size is substantial for a longitudinal study spanning the prenatal period to 12 months of age, the cohort was relatively socially homogeneous. As a result, variability in maternal educational and demographic backgrounds was limited. Future studies including larger, more socioeconomically and demographically diverse samples and allowing for a more robust examination of sex-specific associations will be important to further assess the generalizability of the present findings. Moreover, the assessment of hormones in newborn hair represents a novel and non-invasive approach to capture aspects of the prenatal endocrine environment that are otherwise difficult to access in humans. While this method is inherently challenged by limited hair volume, interindividual variability in hair growth, and the current lack of established reference standards, there were no statistically significant associations of hair length and hair mass with DHEA concentrations in the present study (see **Supplementary Material**, **Supplementary Table 1b**). Although washing procedures were applied to minimize external contamination, the possibility of steroid absorption from amniotic fluid cannot be entirely eliminated (see also 117). Despite these limitations, initial validation work comparing hair-based fetal DHEA measures with cord blood concentrations has yielded promising results (117). While fetal DHEA levels appear to be largely independent of maternal levels (56, 118), we cannot rule out the possibility of maternal influence via intrauterine transfer. As a component of maternal mental health, maternal postpartum mood was assessed using the self-administered EPDS, a validated questionnaire screening instrument for postpartum depression. Although it cannot replace a clinical diagnostic interview (119), the EPDS is considered the most reliable patient-reported screening measure of postpartum depression (120). Future studies could further strengthen the assessment of maternal mental state by using multimodal approaches that combine questionnaire-based measures with clinical interviews and biological markers. These may include endocrine markers such as cortisol (e. g. 121, 122), as well as immune and inflammatory markers (e. g. 123, 124) for a more comprehensive understanding of maternal mood in the postpartum period. This study focuses on maternal mood in the early postpartum period, yet prenatal mood could further contribute to explaining early developmental pathways, as maternal emotional states during pregnancy seem to affect the intrauterine environment, with a focus on maternal prenatal stress in previous research (15, 125). Finally, language assessment at 12 months focused solely on receptive abilities, as infants’ comprehension outpaces their production abilities (126).

Language development is a central domain of early human development. Our findings highlight sex-specific pathways through which biological and environmental factors jointly shape early language outcomes. In boys, higher third-trimester fetal DHEA levels were associated with lower language scores at 12 months, particularly in the context of more favorable postpartum maternal mood, suggesting that developing neural systems may be more responsive to prenatal hormonal signals in supportive environments. In contrast, language development in girls appeared to be primarily sensitive to maternal postpartum mood, independent of fetal hormonal activity. Together, these results underscore the importance of integrating sex-specific prenatal hormonal pathways with early postnatal environmental influences to advance our understanding of early language development and to inform preventive approaches that emphasize parental well-being and support following childbirth.

## Data Availability

The datasets presented in this article are not readily available because of privacy and ethical restrictions. Non-sensitive data are available from the corresponding author upon reasonable request. Requests to access the datasets should be directed to michaela.reimann-ayikoez@plus.ac.at.
