# Supplementary material for: From womb to words: the sex-specific interplay of fetal sex hormones and maternal mood on infant language development
Source: Front Endocrinol (Lausanne). 2026 Jun 16;17:1817292. doi: 10.3389/fendo.2026.1817292 (PMC13314454; doi:10.3389/fendo.2026.1817292)
Supplement: Supplementary file 1 [file Supplementaryfile1.docx]

***Supplementary Materials***

**Evaluation of potential confounding variables in relation to infant receptive language development at 12 months, and fetal DHEA concentrations**

**Table S1a:** Spearman-Correlation Analysis of infant age at 12 months with Bayley-III receptive language scale at 12 months of age (*n* = 58)

| Assessment at 12 months | *Infant age at 12 months* |
| --- | --- |
| Bayley-III: Receptive Language Subscale | ρ = .14, *p =* .30 |

**Table S1b:** Spearman-Correlation Analysis of DHEA concentrations, averaged across *m =* 20 imputed datasets, with hair mass, hair length, and infant age at hair sampling (*n* = 58)

|  | *DHEA* |
| --- | --- |
| Hair length* | ρ = .05, *p =* .69 |
| Hair mass | ρ = .03, *p =* .82 |
| Infant age at hair sample collection | ρ = .12, *p =* .37 |

*Note.* This table presents the spearman correlation results between fetal DHEA and hair mass and length, as well as age at hair sampling, averaged across *m =* 20 imputed datasets, with no statistically significant correlations observed. *The analysis with hair length was performed on *n* = 57 due to one missing value.

**Associations between fetal DHEA, and maternal mood on receptive language development at 12 months of age**

**Table S2a:** Multiple linear regression model examining the main effects of maternal postpartum mood, fetal DHEA levels, and the interaction between fetal DHEA and maternal mood on receptive language abilities at 12 months of age across *m = 20* imputed datasets in boys (*n =* 30), representing the final model selected through backward stepwise regression.

| Variable | Beta | *SE* | β | *p* |
| --- | --- | --- | --- | --- |
| Constant | 18.76 | 1.08 | - | <.001 |
| Fetal DHEA level | -0.19 | 0.05 | -1.03 | <.001 |
| Maternal postpartum mood | -0.52 | 0.19 | -0.70 | .01 |
| Fetal DHEA: Maternal postpartum mood | 0.03 | 0.01 | 0.63 | .03 |

*Note.* This table presents the pooled results from 20 separate multiple linear regression models conducted on *m =* 20 imputed datasets, examining the effect of maternal mood, fetal DHEA levels and their interaction on receptive language abilities at 12 months of age in boys (*pooled R^2^_adj._ across imputations =* .32, *p* < .01).

**Table S2b:** Sensitivity analyses: Final multiple linear regression model examining the main effects of maternal postpartum mood, fetal DHEA levels, and the interaction between fetal DHEA and maternal mood on receptive language abilities at 12 months of age across *m = 20* imputed datasets in boys (*n =* 29)

| Variable | Beta | *SE* | β | *p* |
| --- | --- | --- | --- | --- |
| Constant | 18.77 | 1.10 | - | <.001 |
| Fetal DHEA level | -0.19 | 0.05 | -1.04 | <.001 |
| Maternal postpartum mood | -0.54 | 0.20 | -0.72 | .01 |
| Fetal DHEA: Maternal postpartum mood | 0.03 | 0.01 | 0.65 | .03 |

Note. Of *n =* 30 male participants, *n =* 1 participant was identified as statistical outlier (*SD >* 2.5) and excluded from additional sensitivity analysis. Pooled results of multiple linear regression models on 20 imputed datasets (*pooled R^2^_adj._ across imputations =* .32, *p <* .01).

**Table S3:** Multiple linear regression model examining the main effects of maternal postpartum mood on receptive language abilities at 12 months of age across *m = 20* imputed datasets in girls (*n =* 28), representing the final model selected through backward stepwise regression.

| Variable | Beta | *SE* | β | *p* |
| --- | --- | --- | --- | --- |
| Constant | 16.72 | 0.65 | - | <.001 |
| Maternal postpartum mood | -0.44 | 0.17 | -0.45 | .02 |

*Note.* This table presents the pooled results from 20 separate multiple linear regression models conducted on *m =* 20 imputed datasets, examining the effect of maternal mood on receptive language abilities at 12 months of age in girls (*pooled R^2^_adj._ across imputations =* .17, *p* = .02). As no cases with standardized residuals beyond *2.5 SD* were identified in any of the imputed regression models, sensitivity analysis was not warranted.
